# Broad-Spectrum Disease Resistance Conferred by the Overexpression of Rice RLCK BSR1 Results from an Enhanced Immune Response to Multiple MAMPs

**DOI:** 10.3390/ijms20225523

**Published:** 2019-11-06

**Authors:** Yasukazu Kanda, Hitoshi Nakagawa, Yoko Nishizawa, Takashi Kamakura, Masaki Mori

**Affiliations:** 1 Institute of Agrobiological Sciences, NARO (NIAS), Tsukuba 305-8602, Japan; kanday@affrc.go.jp (Y.K.); nakagawa_hitoshi@mac.com (H.N.); ynishi@affrc.go.jp (Y.N.); 2 Graduate School of Science and Technology, Tokyo University of Science, Noda 278-8510, Japan; kamakura@rs.noda.tus.ac.jp

**Keywords:** disease resistance, microbe-associated molecular pattern (MAMP), *Pyricularia oryzae* (formerly *Magnaporthe oryzae*), *Oryza sativa* (rice), receptor-like cytoplasmic kinase (RLCK), reactive oxygen species (ROS)

## Abstract

Plants activate their immune system through intracellular signaling pathways after perceiving microbe-associated molecular patterns (MAMPs). Receptor-like cytoplasmic kinases mediate the intracellular signaling downstream of pattern-recognition receptors. BROAD-SPECTRUM RESISTANCE 1 (BSR1), a rice (*Oryza sativa*) receptor-like cytoplasmic kinase subfamily-VII protein, contributes to chitin-triggered immune responses. It is valuable for agriculture because its overexpression confers strong disease resistance to fungal and bacterial pathogens. However, it remains unclear how overexpressed BSR1 reinforces plant immunity. Here we analyzed immune responses using rice suspension-cultured cells and sliced leaf blades overexpressing BSR1. BSR1 overexpression enhances MAMP-triggered production of hydrogen peroxide (H_2_O_2_) and transcriptional activation of the defense-related gene in cultured cells and leaf strips. Furthermore, the co-cultivation of leaves with conidia of the blast fungus revealed that BSR1 overexpression allowed host plants to produce detectable oxidative bursts against compatible pathogens. BSR1 was also involved in the immune responses triggered by peptidoglycan and lipopolysaccharide. Thus, we concluded that the hyperactivation of MAMP-triggered immune responses confers BSR1-mediated robust resistance to broad-spectrum pathogens.

## 1. Introduction

Plants combat pathogens by activating their innate immunity. Microbe/pathogen-associated molecular patterns (MAMPs/PAMPs), which have molecular structures that are conserved in fungi or bacteria, alert plants to pathogen attacks. Chitin (a backbone of fungal cell walls), peptidoglycan (a component of bacterial cell walls), lipopolysaccharide (LPS; a component of the outer membranes of Gram-negative bacteria), flagellin, and elongation factor-Tu (EF-Tu) are well-known MAMPs [1]. MAMPs are perceived by corresponding pattern-recognition receptors (PRRs) on host cell surfaces [2]. In rice (*Oryza sativa*), CHITIN ELICITOR RECEPTOR KINASE 1 (OsCERK1), a lysine motif (LysM)-receptor-like kinase (RLK), is well-characterized as a protein component of PRRs. OsCERK1 interacts with a receptor-like protein CHITIN ELICITOR BINDING PROTEIN (CEBiP) [3] to recognize the chitin oligomer [4,5,6] and with rice LYSM-CONTAINING PROTEIN 4/6 (OsLYP4/6) to recognize peptidoglycan [7]. OsCERK1 also functions as a receptor/co-receptor for LPS signaling [8]. Thus, the central RLKs, like OsCERK1, function as hub receptors. PRR complexes activate pattern-triggered immunity (PTI) through intracellular signaling pathways. Protein phosphorylation signals originating in PRRs positively regulate early phase responses, such as oxidative burst and the activation of mitogen-activated protein kinase (MAPK) cascades, followed by the transcriptional activation of defense-related genes [9,10]. Oxidative burst is caused by a rapid production of reactive oxygen species (ROS) by plant NADPH oxidases, called RESPIRATORY BURST OXIDASE HOMOLOG (RBOH) proteins [11]. Host-derived ROS play various roles in PTI. They possess an antimicrobial activity that can kill microbes, and they enhance physical barrier production by promoting lignin synthesis and cross-linking of plant cell walls [12,13,14,15]. Hydrogen peroxide (H_2_O_2_), an ROS produced in oxidative bursts, acts as a signaling molecule to induce the transcriptional activation of defense-related genes, biosynthesis of phytoalexin, and programmed cell death [11,16].

Pathogens have adapted to suppress PTI through the secretion of effectors using type III secretion system (TTSS) and structural variations on MAMPs [17]. Molina and Kahmann (2007) reported that the detoxification of host-derived ROS is required for a biotrophic pathogen of maize *Ustilago maydis* to overcome PTI [18]. The deletion of *YAP*, encoding an oxidative stress-responsive transcription factor, in *U. maydis* increases the sensitivity to H_2_O_2_ and significantly decreases the pathogenicity. Rice blast fungus (*Pyricularia oryzae*), which causes a serious disease in rice, releases a catalase-peroxidase B (CPXB)-dependent ROS-degrading activity near conidia [19,20]. Enzymes that compose the glutathione and thioredoxin antioxidation system in *P. oryzae* are required for virulence as well as resistance to ROS [21,22]. *P. oryzae* mutant strains Δ*des1* and Δ*sir2*, which lack transcriptional regulators for extracellular peroxidases and superoxide dismutase, cannot form susceptible lesions because they induce defense responses, including the accumulation of host-derived ROS and the upregulation of defense-related genes [23,24]. These findings indicate that host-derived ROS is a crucial factor in host–microbe interactions.

Receptor-like cytoplasmic kinases (RLCKs) contribute to cytoplasmic phosphorylation signaling pathways in PTI. RLCKs are characterized as cytoplasmic proteins that contain a RLK-homologous kinase domain but not a transmembrane domain. *Arabidopsis thaliana* and rice have 147 and 379 RLCK-encoding genes, respectively [25,26]. RLCKs are classified into 17 subfamilies based on their sequence features. Several RLCKs belonging to subfamily-VII are involved in PTI [27]. In *A. thaliana*, BOTRYTIS-INDUCED KINASE 1 (BIK1) is phosphorylated by PRRs composed of BRI1-ASSOCIATED KINASE 1 (BAK1) and FLAGELLIN SENSING 2 (FLS2) or EF-Tu RECEPTOR (EFR) depending on the ligand (flagellin or EF-Tu, respectively)-binding [28,29]. Phosphorylated BIK1 further phosphorylates and activates RBOHD, which is responsible for the oxidative bursts in PTI [30,31]. In rice, OsRLCK176, an ortholog of BIK1, interacts with OsCERK1 to mediate chitin- and peptidoglycan-induced defense responses [7]. OsRLCK57, OsRLCK107, and OsRLCK118, which are highly homologous to OsRLCK176, also have similar functions [32]. OsRLCK118 directly phosphorylates OsRBOHB [33]. OsRLCK185 interacts with OsCERK1 and MAPKKK, connecting PRRs and the MAPK cascade [34,35].

BROAD-SPECTRUM RESISTANCE 1 (BSR1; OsRLCK278), a rice RLCK-VII protein, has unique disease control effects when overexpressed. It was identified in a screen for disease resistance in rice Full-length cDNA OvereXpressor (FOX) *Arabidopsis* lines [36,37]. The screening revealed that transgenic *A. thaliana* plants overexpressing BSR1 were highly resistant to *Pseudomonas syringae* pv. *tomato* DC3000 and *Colletotrichum higginsianum*. Furthermore, overexpression of BSR1 in rice conferred strong resistance against four rice pathogens: rice blast fungus, brown spot fungus (*Cochliobolus miyabeanus*), rice leaf blight bacteria (*Xanthomonas oryzae* pv. *oryzae*), and *Burkholderia glumae*, which is the causal agent of bacterial seedling rot and bacterial grain rot [36,38]. To our knowledge, among the many RLCKs, BSR1 is the only one that can enhance disease resistance when overexpressed. However, the mechanism underlying the broad-spectrum disease resistance conferred by the overexpression of BSR1 remains unknown.

The contribution of BSR1 to the innate immunity of wild-type rice has been analyzed. A knockout of *BSR1* caused significant suppression of chitin-induced defense-responses, including oxidative bursts and the transcriptional activation of defense-related genes [39]. BSR1 has an active protein kinase domain that phosphorylates serine/threonine and tyrosine residues [40]. These indicate that BSR1 should mediate the downstream phosphorylation signaling of OsCERK1, because the perception of chitin completely depends on OsCERK1 [4]. The silencing of *BSR1* decreased resistance to not only fungal but also bacterial diseases [40], suggesting that BSR1 is involved in the signaling pathway activated by bacterial MAMPs downstream of OsCERK1.

In this report, we investigated whether BSR1 contributes to defense responses elicited by bacterial MAMPs. The resulting resistance is almost independent of salicylic acid, a plant hormone related to immunity [40]. Therefore, we focused on the early phase of defense responses, like the oxidative bursts. Furthermore, to reveal the mechanisms underlying broad-spectrum disease resistance in the BSR1-overexpressing rice plants, we analyzed the early defense events using suspension-cultured cells and sliced leaf blades overexpressing BSR1.

## 2. Results

### 2.1. BSR1 Contributes to Bacterial MAMP-Induced Oxidative Bursts

**Figure 1:**
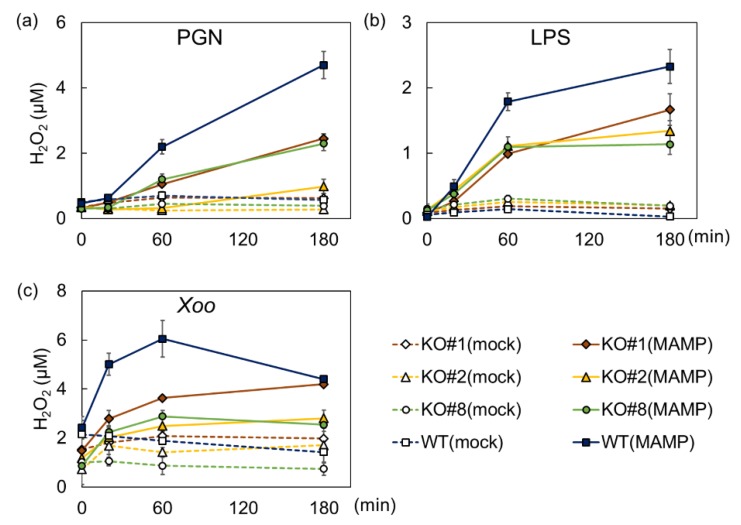
Knockouts of *BSR1* impaired H_2_O_2_ production in rice cell cultures treated with MAMPs. Suspension-cultured cells were treated with peptidoglycan (**a**), LPS (**b**), or an autoclaved suspension of *Xanthomonas oryzae* pv. *oryzae* (*Xoo*; **c**). H_2_O_2_ concentrations were measured before treatment and at 20, 60, and 180 min after treatment. Values are presented as the means ± standard deviations of three biological replicates. Experiments were conducted twice with similar results. PGN, peptidoglycan; LPS, lipopolysaccharide; KO, knockout line; KO#1, *bsr1-1*#13-1; KO#2, *bsr1-2*#16-2; KO#8, *bsr1-8*#5-1; WT, wild-type; MAMPs, microbe-associated molecular patterns; BSR1, BROAD-SPECTRUM RESISTANCE 1. The statistical analysis was performed as shown in Supplementary Materials Table S2.

To assess the contribution of BSR1 to bacterium-derived MAMP-induced defense responses, we evaluated the effects of BSR1 knockout on defense responses using three independent *BSR1*-knockout lines. These lines were generated in our previous study and contain homozygous frameshift mutations in exon 1 of *BSR1* [39]. Suspension-cultured cells were derived from knockout and non-transgenic (wild-type) lines and treated with the bacterium-derived MAMPs peptidoglycan and LPS. After treatment with peptidoglycan, suspension-cultured cells derived from all three *BSR1*-knockout lines produced lower H_2_O_2_ concentrations than wild-type cells (Figure 1a; Supplementary Materials Table S2a). At 180 min after addition, 59%–71% of H_2_O_2_ production was lost in knockout cells. The LPS treatment also induced impaired oxidative bursts in *BSR1*-knockout cells (Figure 1b; Supplementary Materials Table S2b). These cells accumulated 38%–45% lower amounts of H_2_O_2_ compared with wild-type at 60 min after treatment. Knockout mutations in *BSR1* significantly suppressed the oxidative bursts but they were not completely abolished, indicating functional redundancy for BSR1. Thus, BSR1 plays a role in the induction of oxidative bursts in response to peptidoglycan and LPS.

To further provide support for the involvement of BSR1 in oxidative bursts against bacterial infections, autoclaved *X. oryzae* pv. *oryzae* cells were used as the elicitor. Knocking out *BSR1* reduced the production of H_2_O_2_ by 39%–58% at 60 min after treatment with the autoclaved cells (Figure 1c; Supplementary Materials Table S2c). Thus, BSR1 should contribute to defense responses against not only MAMPs purified from nonpathogenic microbes but also against the cellular components of pathogenic bacteria.

### 2.2. BSR1 Is Involved in Regulating MAMP-Responsive Genes

**Figure 2:**
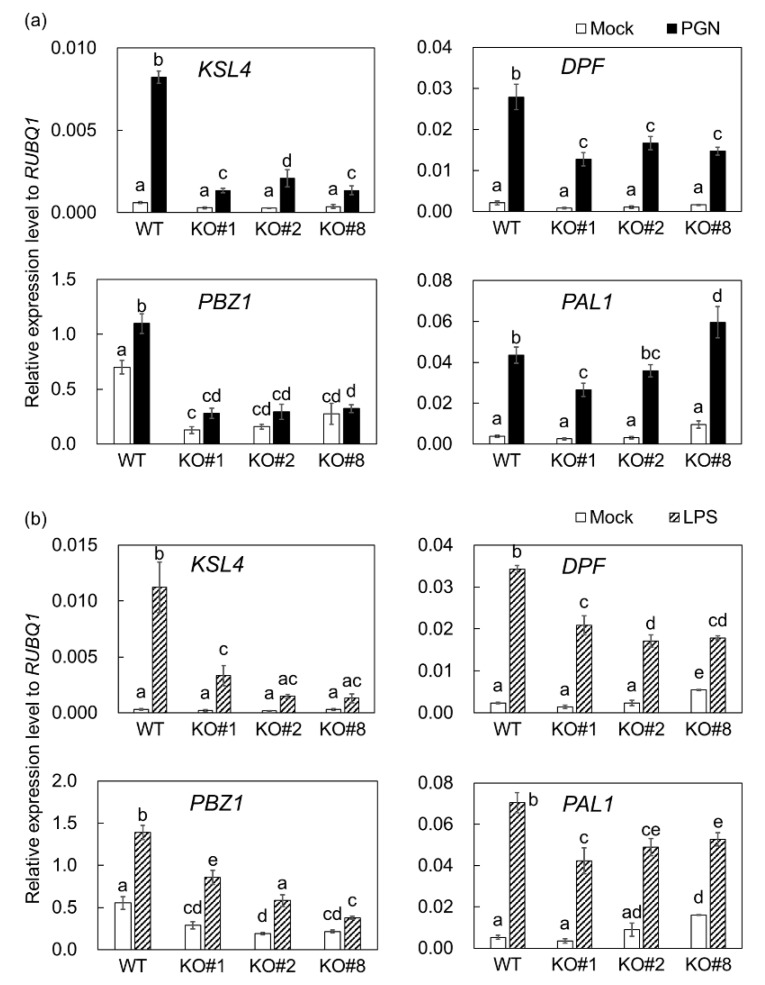
MAMP-induced transcript levels of defense-related genes were suppressed in *BSR1*-knockout suspension-cultured rice cells. The *PBZ1*, *PAL1*, *KSL4*, and *DPF* transcript levels at 3-h post treatment with peptidoglycan (**a**) and LPS (**b**) were normalized against the *RUBQ1* internal control levels. Values are presented as the means ± standard deviations of three biological replicates. Experiments were conducted two times with similar results. Different letters indicate significant differences (Tukey’s test; *p* < 0.05). PGN, peptidoglycan; KO, knockout line; KO#1, *bsr1-1*#13-1; KO#2, *bsr1-2*#16-2; KO#8, *bsr1-8*#5-1; WT, wild-type; LPS, lipopolysaccharide.

In MAMP-treated suspension-cultured cells, the transcriptional activation of defense-related genes was analyzed. Transcript levels of four defense-related genes, diterpenoid phytoalexin (DP) momilactone biosynthetic gene *KAURENE SYNTHASE-LIKE 4* (*KSL4*), DP biosynthetic key transcription factor-encoding gene *DITERPENOID PHYTOALEXIN FACTOR* (*DPF*) [41], the representative defense marker gene *PROBENAZOLE-INDUCIBLE PROTEIN 1* (*PBZ1*), and flavonoid phytoalexin and lignin biosynthetic gene *PHENYLALANINE AMMONIA-LYASE 1* (*PAL1*) were determined. After treatment with peptidoglycan, the inductions of *KSL4*, *DPF*, and *PBZ1* in knockout cells were significantly weaker than in wild-type, although significant changes in *PAL1* transcript level were not detected (Figure 2a). Knocking out *BSR1* resulted in a decrease in *PBZ1* transcript levels under mock-treatment conditions (Figure 2). Our liquid cultivation conditions slightly induced *PBZ1* transcriptional activation, which was mediated by BSR1.

Knocking out *BSR1* suppressed the elicitation of *KSL4* and *DPF* by LPS (Figure 2b). The significant suppression of *PBZ1* and *PAL1* were not reproducibly detected. As shown in Figure 1 and Figure 2, BSR1 appears to function in defense responses after the plant perceives peptidoglycan and LPS.

### 2.3. BSR1 Overexpression Enhances Oxidative Bursts in Suspension-Cultured Cells

Contrary to the *BSR1* disruption phenotype, the overexpression of BSR1 is assumed to enhance defense responses. Whether the overexpression affects the robustness of the oxidative bursts and transcriptional activation was investigated. Rice plants overexpressing HA–PreScission–Biotin (HPB)-tagged BSR1 and GUS (BSR1-HPB:OX and GUS-HPB:OX, respectively) were generated. The GUS-HPB:OX line was used as a control. The integrities of the inserted constructs were confirmed by western analysis with an anti-HA antibody (Supplementary Materials Figure S1a). The overexpression of BSR1-HPB conferred resistance to rice blast, indicating that BSR1-HPB is functional (Supplementary Materials Figure S1b). Suspension-cultured cells were prepared from wild-type, GUS-HPB:OX, and two independent BSR1-HPB:OX lines. The transcript levels of *BSR1* and HPB-tagged transgenes in suspension-cultured cells were ascertained using qRT-PCR (Supplementary Materials Figure S1c).

**Figure 3:**
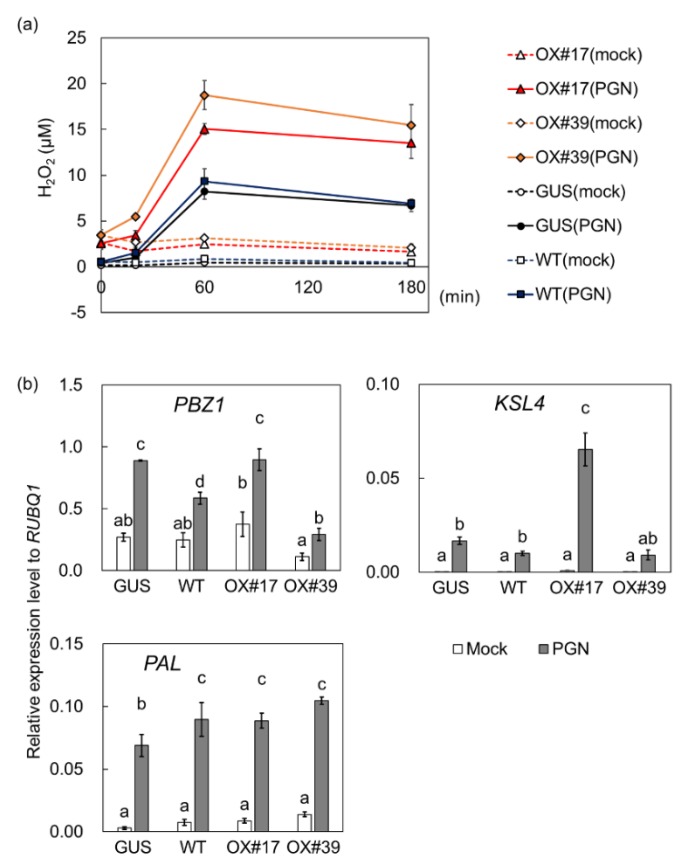
The overexpression of BSR1-HPB enhanced peptidoglycan-induced oxidative bursts in suspension-cultured rice cells. Cells treated with peptidoglycan were analyzed for H_2_O_2_ production accompanying oxidative bursts (**a**) and the transcript levels of defense-related genes (**b**). Values are presented as the means ± standard deviations of three biological replicates. In (**a**), H_2_O_2_ concentrations were measured before treatment and at 20, 60, and 180 min after treatment. The statistical analysis was performed as shown in Figure S2a. Experiments were conducted three times with similar results. In (**b**), the *PBZ1*, *PAL1*, and *KSL4* transcript levels were normalized against the *RUBQ1* internal control levels. Experiments were conducted two times with similar results. Different letters indicate significant differences (Tukey’s test; *p* < 0.05). PGN, peptidoglycan; OX, overexpressing line; HPB, HA–PreScission–Biotin; OX#17, BSR1-HPB:OX#17; OX#39, BSR1-HPB:OX#39; GUS, GUS-HPB:OX; WT, wild-type.

In response to peptidoglycan treatments, suspension-cultured cells derived from two BSR1-HPB:OX lines produced H_2_O_2_ more rapidly than GUS-HPB:OX (Figure 3a; Supplementary Materials Figure S2a). At 60 min after treatment, the overexpression of BSR1 resulted in increased H_2_O_2_ concentrations to 1.6–2.0 times that of the control (Figure 3a). Transcript level of a defense-related gene *PAL1* was increased in BSR1-HPB:OX cells compared with GUS-HPB:OX control, while no significant changes in transcript levels of *PBZ1* and *KSL4* were detected (Figure 3b). Transcript levels in GUS-HPB:OX did not necessarily agree with those in WT, indicating that the responses would be slightly altered by overexpression of transgenes. BSR1-HPB:OX cells produced enhanced H_2_O_2_ bursts in response to LPS as well as peptidoglycan (Supplementary Materials Figure S3).

**Figure 4:**
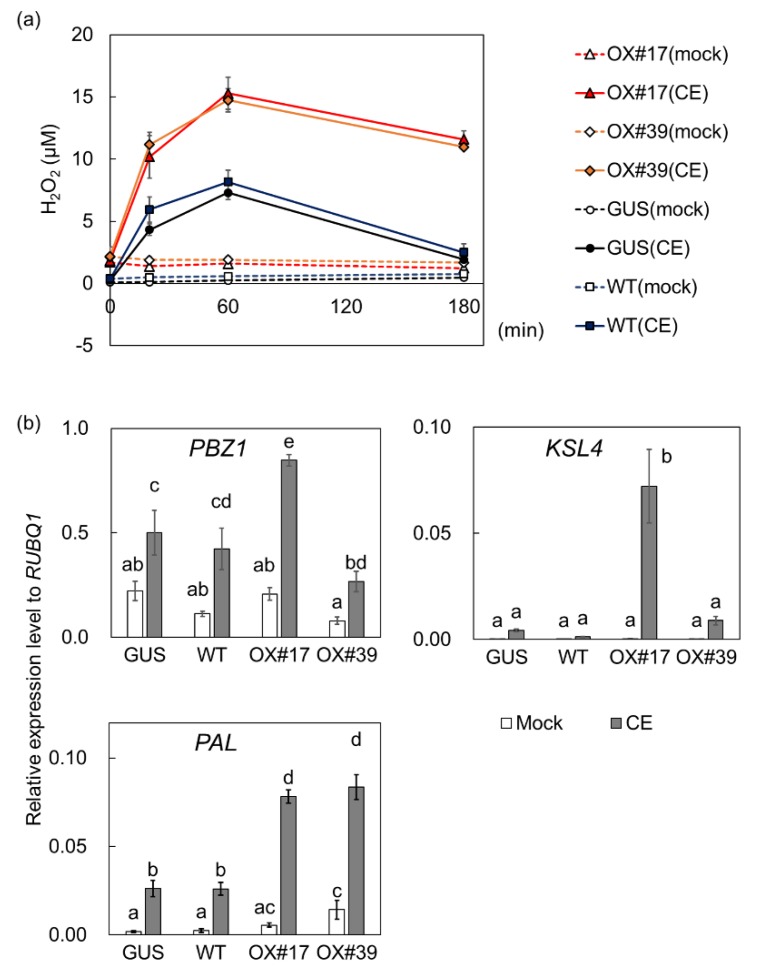
The overexpression of BSR1-HPB enhanced chitin-induced defense responses in suspension-cultured rice cells. Values are presented as the means ± standard deviations of three biological replicates. (**a**) H_2_O_2_ concentrations were measured before treatment and at 20, 60, and 180 min after treatment. The statistical analysis was performed as shown in Figure S2b. Experiments were conducted three times with similar results. (**b**) The *PBZ1*, *PAL1*, and *KSL4* transcript levels were normalized against the *RUBQ1* internal control levels. Experiments were conducted twice with similar results. Different letters indicate significant differences (Tukey’s test; *p* < 0.05). CE, chitin elicitor; OX, overexpressing line; HPB, HA–PreScission–Biotin; OX#17, BSR1-HPB:OX#17; OX#39, BSR1-HPB:OX#39; GUS, GUS-HPB:OX; WT, wild-type.

Interestingly, before the MAMP treatment, the overexpression of BSR1-HPB resulted in a slight but statistically significant increase in H_2_O_2_ concentrations compared with GUS-HPB in cell cultures. Comparisons between the untreated conditions (Figure 3a, 0 min) showed that there were significant differences between BSR1-HPB:OX lines and the GUS-HPB:OX line (*p* < 0.001 for BSR1-HPB:OX17 and BSR1-HPB:OX39, Student’s *t*-test). These phenotypes were common to all the replicated experiments (Figure 4; Supplementary Materials Figure S3). BSR1 overexpression did not increase transcript levels of *RbohB*, encoding a NADPH oxidase related to ROS burst (Supplementary Materials Figure S4). These results suggest that an excess of BSR1 protein could constitutively promote NADPH oxidase activity of RBOH proteins but not their transcription.

Because of requirement of BSR1 in chitin oligomer-induced defense responses [39], we assessed the oxidative bursts after a chitin hexamer treatment. The amount of H_2_O_2_ produced by BSR1-HPB:OX cells significantly exceeded that of the control at each measured time point (Figure 4a; Supplementary Materials Figure S2b). At 60 min after treatment, BSR1-HPB:OX cells produced a 1.8–1.9-fold greater H_2_O_2_ concentration than GUS-HPB:OX cells (Figure 4a). The chitin-induced transcriptional activation of *PAL1*, but not *KSL4* and *PBZ1*, were enhanced by the overexpression of BSR1-HPB (Figure 4b). These comparisons of BSR1-HPBs with GUS-HPB clearly showed that BSR1 overexpression enhanced oxidative bursts and transcriptional activation of, at least, *PAL1* in response to multiple MAMPs.

### 2.4. Oxidative Bursts against Blast Fungus Are Enhanced in Plants Overexpressing BSR1

**Figure 5:**
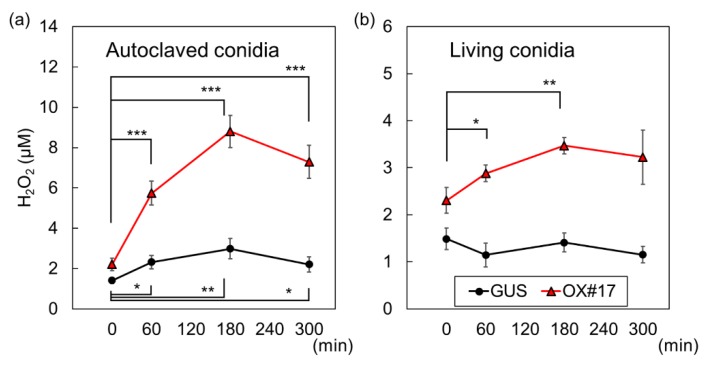
Rice leaf strips derived from BSR1-HPB:OX plants caused an enhanced burst of H_2_O_2_ when exposed to conidia of the compatible blast fungus. Leaf strips were cultivated with 8 × 10^4^ mL^−1^ autoclaved conidia (**a**) or 8 × 10^3^ mL^−1^ living conidia (**b**) in wells of a 12-well plate. H_2_O_2_ concentrations in wells were measured before treatment and at 60, 180, and 300 min after treatment. Values are presented as the means ± standard deviations of three biological replicates. Asterisks indicate significant differences between the untreated condition (0 min) values and the values at the indicated times in the same line (Student’s *t*-test; * *p* < 0.05, ** *p* < 0.01, and *** *p* < 0.001). Experiments were conducted twice with similar results. OX, overexpressing line; HPB, HA–PreScission–Biotin; OX#17, BSR1-HPB:OX#17; GUS, GUS-HPB:OX.

We speculated that H_2_O_2_ production in plant leaves as a response to pathogen challenges is increased by the overexpression of BSR1, as observed in suspension-cultured cells. To test the hypothesis, strips from leaf blades were quantitatively analyzed for H_2_O_2_ production after being treated with conidia of the blast fungus, which had been autoclaved to eliminate any biological activity. Before the treatment, the H_2_O_2_ concentration in water containing leaf strips of BSR1-HPB:OX#17 was slightly greater than that of GUS-HPB:OX (Figure 5). After exposure to autoclaved conidia, leaf strips of BSR1-HPB:OX plants produced far greater H_2_O_2_ concentrations than those of GUS-HPB:OX plants (Figure 5a). Taking into consideration the difference between untreated conditions, we calculated changes in H_2_O_2_ concentrations during the experiment. By 180 min after treatment, the overexpression of BSR1-HPB resulted in a ~4.2-fold increase in changes in H_2_O_2_ concentration, compared with GUS-HPB (Supplementary Materials Figure S5a). Autoclaved conidia-induced H_2_O_2_ hyperproduction was also detected in leaf strips of BSR1-HPB:OX#39, an another BSR1-overexpressing line (Supplementary Materials Figure S6). Thus, *BSR1* overexpression enhanced oxidative bursts in leaf blades.

To assess the importance of the enhanced H_2_O_2_ bursts in host–microbe interactions, we also examined the oxidative bursts after a living conidia treatment. A treatment with 8 × 10^4^ mL^−1^ conidia depressed H_2_O_2_ levels in BSR1-HPB:OX leaves and GUS-HPB:OX leaves (Supplementary Materials Figure S7). This result corroborated a previous report that suspensions of conidia contain H_2_O_2_-degrading enzymes [19]. In order to avoid that abnormally strong ROS-degrading activity obscures the difference, we used lower concentration (8 × 10^3^ mL^−1^) of conidia. Considering that the H_2_O_2_-degrading activity increased with the conidial concentration, comparisons between H_2_O_2_ levels were performed only under the same co-cultivation conditions. When co-cultivated with 8 × 10^3^ mL^−1^ conidia, no elevation in the H_2_O_2_ level was detected in GUS-HPB:OX leaves (Figure 5b; Supplementary Materials Figure S5b). Thus, the addition of this concentration of conidia completely suppressed MAMP-induced oxidative bursts in the control line. In contrast, when co-cultivated with BSR1-HPB:OX leaves, the H_2_O_2_ level significantly increased, compared with before the conidial inoculation (Figure 5b). These co-cultivation experiments revealed that rice plants overexpressing BSR1 produced large amounts of H_2_O_2_ that overwhelmed the ROS degradation caused by pathogens.

## 3. Discussion

**Figure 6:**
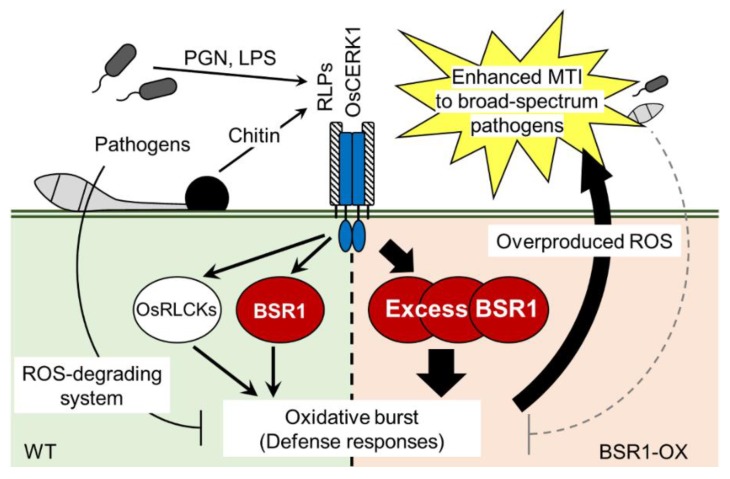
Proposed model in which BSR1 regulates defense responses, such as oxidative bursts, after the perception of MAMPs in wild-type (WT; **left**) and BSR1-overexpressing rice lines (BSR1-OX; **right**). PGN, peptidoglycan; LPS, lipopolysaccharide; RLPs, receptor-like proteins; ROS, reactive oxygen species; MTI, MAMP-triggered immunity.

BSR1, a RLCK-VII member, has a protein kinase activity and is important for the initiation of defense responses against chitin oligomers, known as a fungus-derived MAMP [39,40]. BSR1 is implicated in resistance to bacteria, as well as fungi, in wild-type and overexpressing rice lines [36,38,40], suggesting that BSR1 is also involved in responses triggered by bacterium-derived MAMPs. In this study, our experiments on suspension-cultured rice cells showed a correlation between BSR1 and the response to two bacterial elicitors, peptidoglycan and LPS. Knocking out *BSR1* significantly suppressed the elicitation of oxidative bursts and the transcript levels of defense-related genes caused by these bacterial MAMPs (Figure 1 and Figure 2). In some experiments, variations of the H_2_O_2_ concentration and the transcript levels were observed among knockout lines (Figure 1 and Figure 2). Since the variations among knockout lines were not reproducible, they were considered as the influence of the experimental manipulation. The suppression of immune responses by *BSR1* knockout is in accordance with the significant contribution of BSR1 to chitin-induced responses [39]. Rice recognizes chitin through receptor complexes containing OsCERK1 and CEBiP [6]. OsCERK1, but not CEBiP, possesses a protein kinase activity to phosphorylate cytoplasmic signaling factors [42]. The perception of peptidoglycan and LPS was mostly mediated by OsCERK1 complex [4,7,8]. Thus, in response to peptidoglycan and LPS exposure, as well as chitin, OsCERK1 would transmit a signal directly or indirectly to BSR1 to regulate its protein kinase activity (Figure 6).

Knocking out *BSR1* did not make rice cells nonresponsive to MAMPs (Figure 1 and Figure 2), indicating the existence of functionally redundant factor(s) for BSR1. In *A. thaliana*, RLCK-VII members function in MAMP-induced defense responses with a robust functional redundancy [43]. The participation of other rice RLCK-VII members in PTI have been studied [27]. OsRLCK57, OsRLCK107, OsRLCK118, OsRLCK176, and OsRLCK185 positively regulate chitin- and peptidoglycan-induced responses [7,32,42]. No rice RLCKs, except for BSR1, have been reported to mediate LPS-induced oxidative bursts. However, known interactors of the LPS-(co)receptor OsCERK1, such as OsRLCK176 and OsRLCK185, may mediate LPS-signaling. To take into consideration of functional redundancy for BSR1, these RLCK-VII members could act downstream of LPS as well as peptidoglycan.

We compared BSR1-HPB:OX lines with GUS-HPB:OX control line to assess the effects of BSR1 overexpression on MAMP-triggered responses. In the absence of MAMPs, H_2_O_2_ levels in cell cultures and leaf strips derived from BSR1-HPB:OX lines were slightly greater than those of the control (Figure 3, Figure 4 and Figure 5). Where this H_2_O_2_ originates from is unknown. Under peptidoglycan-, LPS-, and chitin-treated conditions, BSR1-HPB:OX suspension-cultured cells produced a greater amount of H_2_O_2_ than control cells (Figure 3 and Figure 4; Supplementary Materials Figures S2 and S3), while the overexpression of BSR1 facilitated the transcriptional activation of *PAL1* but not *PBZ1* and *KSL4* (Figure 3b and Figure 4b). There were variations in transcript levels of *PBZ1* and *KSL4* between two BSR1 overexpression lines, BSR1-HPB:OX#17 and BSR1-HPB:OX#39. Since transcript levels of defense-marker gene *PBZ1* in MAMP-treated and untreated BSR1-HPB:OX#39 were even lower than those in the control line, the line may have contained mutations which decrease the transcript levels of these defense-related genes. Alternatively, the condition to culture BSR1-HPB:OX#39 line may have given stress to the cells, resulting in slight increase in the expression of internal control *RUBQ1*, which encodes protein turnover factor. That is because the environment surrounding the cells, such as cell concentration, cannot be completely uniformized. In accordance with results using cell culture, BSR1-HPB:OX leaf blade tissues displayed remarkably greater oxidative bursts against MAMPs extracted from autoclaved conidia (Figure 5a). In rice and *Arabidopsis*, OsRLCK118 and *A. thaliana* BIK1, two RLCK-VII members, directly and positively regulate RBOH proteins whose NADPH oxidase activities generate ROS and cause oxidative bursts [30,31,33]. Highly expressed BIK1 leads to enhanced ROS production in response to MAMP [44]. Excess BSR1 protein also could hyperactivate RBOHs, directly or indirectly, resulting in the enhancement of oxidative bursts. Recent work showed that *BIK1* overexpression does not enhance fungal disease resistance, although deletions of negative regulators for PTI signaling result in the accumulation of BIK1 and the strong disease resistance in *A. thaliana* [45]. Unlike BIK1, BSR1 overexpression confers the robust disease resistance in rice and *A. thaliana* [36], indicating that functions of BSR1 would be quite different from those of BIK1.

A time course of the H_2_O_2_ levels under co-cultivation conditions revealed how the overexpression of BSR1 acts during the early phase of host–microbe interactions. Under our co-cultivation conditions, suspensions of living conidia of the blast fungus did not elicit host-derived H_2_O_2_ production in control leaf strips (Figure 5b; Supplementary Materials Figure S5b). These results reconfirmed previous reports that the supernatants of conidial suspensions contain ROS-degrading activities that mostly depend on CPXB, a catalase-peroxidase secreted by the blast fungus [19]. Under the same co-cultivation conditions, the overexpression of BSR1-HPB resulted in leaf blade tissues producing detectable amounts of H_2_O_2_ (Figure 5), indicating that oxidative bursts in BSR1-overexpressing plants are intense enough to overcome the inhibition caused by the infecting blast fungus. The enhanced responses against peptidoglycan and LPS, as well as chitin resulting from the overexpression (Figure 3 and Figure 4; Supplementary Materials Figure S3) strongly suggested that pathogenic bacterial challenges would elicit the same responses.

In host–microbe interactions, host-derived ROS is regarded as an antimicrobial substance and a diffusible second messenger that contributes to immunity [11,16]. Indeed, host-derived ROS detoxification should be required for pathogenicity. For example, ROS-degrading activities are present in the supernatants of *P. oryzae* conidial suspensions and contribute to lesion formation when exogenously added [20]. The deletion of *P. oryzae DES1*, which is required for extracellular peroxidase activity, causes the accumulation of host-derived ROS and the induction of defense-related genes, resulting in non-pathogenicity [23]. Pathogens could not completely abolish overproduced ROS (Figure 5b), and therefore do not show full virulence in plants overexpressing BSR1 (Figure 6). Our data support the model which host-derived ROS is critical for plant interactions with pathogens.

In conclusion, we propose that the broad-spectrum disease resistance could be achieved by the enhancement of MAMP-triggered oxidative bursts and following transcriptional activation. To date, many RLCKs have been characterized as signaling factors in PTI [27]. However, no RLCK, other than *BSR1*, can enhance oxidative bursts and disease resistance when overexpressed in rice. It is unclear what allows the functional enhancement. This report clearly showed that hyperactivated MAMP-triggered immune responses could be used for broad-spectrum disease control.

## 4. Materials and Methods

### 4.1. Plant and Microbial Materials and Inoculation

Rice (*Oryza sativa* L. cv. Nipponbare) was used as the wild-type (WT) plant material. The *BSR1*-knockout lines *bsr1-1*#13-1 (KO#1), *bsr1-2*#16-2 (KO#2), and *bsr1-8*#5-1 (KO#8) generated with the clustered regularly interspaced short palindromic repeats (CRISPR)/CRISPR-associated 9 (Cas9) system in our previous study [39,46] were used. Rice calli were prepared from dehusked seeds and cultivated on N6D medium containing 0.4% gellan gum [47]. The *Pyricularia oryzae* isolate Kyu89-246 (MAFF101506, race 003.0), which is compatible with Nipponbare rice plants, was used to prepare the elicitor and for inoculations. *Xanthomonas oryzae* pv. *oryzae* (isolate T7174) was used to prepare the elicitor fraction. The culturing methods and *P. oryzae* and *X. oryzae* pv. *oryzae* inoculation techniques were as previously described [38].

### 4.2. Plasmid Construction and Transformation

A DNA fragment containing the maize *Ubiquitin-1* promoter was excised from pRiceFOX-GateA-*SG1* [48] with HindIII and KpnI. pMDC32-HPB [49] was digested with HindIII and KpnI, and the fragment containing the 2× 35S promoter was replaced with the HindIII–KpnI fragment containing the maize *Ubiquitin-1* promoter. The resulting plasmid was named pMDC32-Mubi-HPB. The open reading frame sequences of *BSR1* and *GUS* were amplified from the full-length cDNA (AK070024) and pBI221 (AF502128), respectively. These DNA fragments were ligated into pENTR/D-TOPO (Invitrogen, Carlsbad, CA, USA) to construct HPB-tagged *BSR1* and *GUS* (*BSR1-HPB* and *GUS-HPB*, respectively) in pMDC32-Mubi-HPB using the Gateway LR Clonase II Plus Enzyme (Invitrogen). pMDC32-Mubi-HPB containing *BSR1-HPB* or *GUS-HPB* was introduced into Nipponbare using the *Rhizobium radiobacter*-mediated transformation method [47]. BSR1-HPB:OX17 (OX#17), BSR1-HPB:OX39 (OX#39), and GUS-HPB:OX6 (GUS) transgenic lines were analyzed as two BSR1-overexpressing lines and a control line, respectively.

### 4.3. Measurement of H_2_O_2_

Rice suspension-cultured cells were prepared using a previously published method [5,39]. Modified liquid N6 medium (30 g L^−1^ sucrose; 4.1 mg L^−1^ N6 salt (Wako, Osaka, Japan); 2 mg L^−1^ glycine; 0.5 mg L^−1^ nicotinic acid; 0.5 mg L^−1^ pyridoxine HCl; 1 mg L^−1^ thiamine HCl; 100 mg L^−1^ myo-inositol; 1 mg L^−1^ 2,4-dichlorophenoxyacetic acid; 23.4 mg L^−1^ MnSO_4_·4H_2_O; pH 5.8) was used for liquid cultivation. We treated 1 mL media containing 100 mg suspension-cultured cells with 10 µg mL^−1^ peptidoglycan from *Bacillus subtilis* (Sigma-Aldrich, St. Louis, MO, USA), 50 µg mL^−1^ LPS from *Pseudomonas aeruginosa* 10 purified by phenol extraction (Sigma-Aldrich), 10 nM *N*-acetylchitohexaose (chitin elicitor (CE)), or an autoclaved suspension of *X. oryzae* pv. *oryzae* (OD_600_ = 0.3).

To prepare leaf strips, the sixth leaves of GUS-HPB:OX and BSR1-HPB:OX17 plants at the 6–6.5-leaf stage were used. Two fragments of the leaf blades (8-mm length and 6-mm width) that were slit using bundled razor blades at approximately 0.5-mm intervals were placed in a well of a 12-well plate. Leaf strips were floated on sterile water and incubated at 28 °C for 14–15 h with shaking at 90 rpm, followed by a 1-h incubation in new water. Conidia of rice blast fungus were scraped from the gel surface with sterile water and filtered through a Kimwipe. The conidial concentration in the filtrate was calculated using a hemocytometer. A suspension of autoclaved or living conidia was poured into the wells to the indicated final concentration and incubated at 28 °C. The H_2_O_2_ concentration was determined at the indicated time using a previously described luminol-dependent chemiluminescence assay [5]. The statistical analyses were carried out using Dunnett’s test for the experiments with cultured cells and Student’s *t*-test for the experiments with leaf strips.

### 4.4. Quantitative Reverse Transcription (qRT)-PCR

Cultured rice cells were frozen in liquid nitrogen after a 3-h treatment with 10 µg mL^−1^ peptidoglycan, 50 µg mL^−1^ LPS, 10 nM *N*-acetylchitohexaose, an autoclaved suspension of *X. oryzae* pv. *oryzae* (OD_600_ = 0.3), or sterile water. Total RNA extraction and the qRT-PCR were performed as previously described [39]. Transcript levels were analyzed using the comparative C_T_ (2^−ΔΔCt^) method with rice *Ubiquitin1* (*RUBQ1*; Os06g0681400) as an internal control [50,51]. The statistical analysis was carried out with Tukey’s test. The primers presented in Supplementary Materials Table S1 were used for the qRT-PCR analyses.

### 4.5. Western Blot Analysis

Protein was extracted from 50 mg leaf blades with 300 µL SDS–urea buffer (8 M urea, 5% SDS, 0.1 mM EDTA, 2% 2-mercaptoethanol, 1 mM phenylmethanesulfonylfluoride (PMSF), 2 × complete inhibitor mix, EDTA-free (Roche, Basel, Switzerland), 40 mM Tris-HCl: pH 6.8) according to a previously described method [52]. An equal volume of each SDS–urea sample was used for the western analysis. HPB-tagged protein was detected using anti-HA antibody (Anti-HA.11, Mouse-Mono 16B12; BAB).

## Supplementary Materials

The following are available online at https://www.mdpi.com/1422-0067/20/22/5523/s1.
