# Supplementary material for: Broad-Spectrum Disease Resistance Conferred by the Overexpression of Rice RLCK BSR1 Results from an Enhanced Immune Response to Multiple MAMPs
Source: Int J Mol Sci. 2019 Nov 6;20(22):5523. doi: 10.3390/ijms20225523 (PMC6888047; doi:10.3390/ijms20225523)
Supplement: Supplementary file 1 [file ijms-20-05523-s001.pdf]

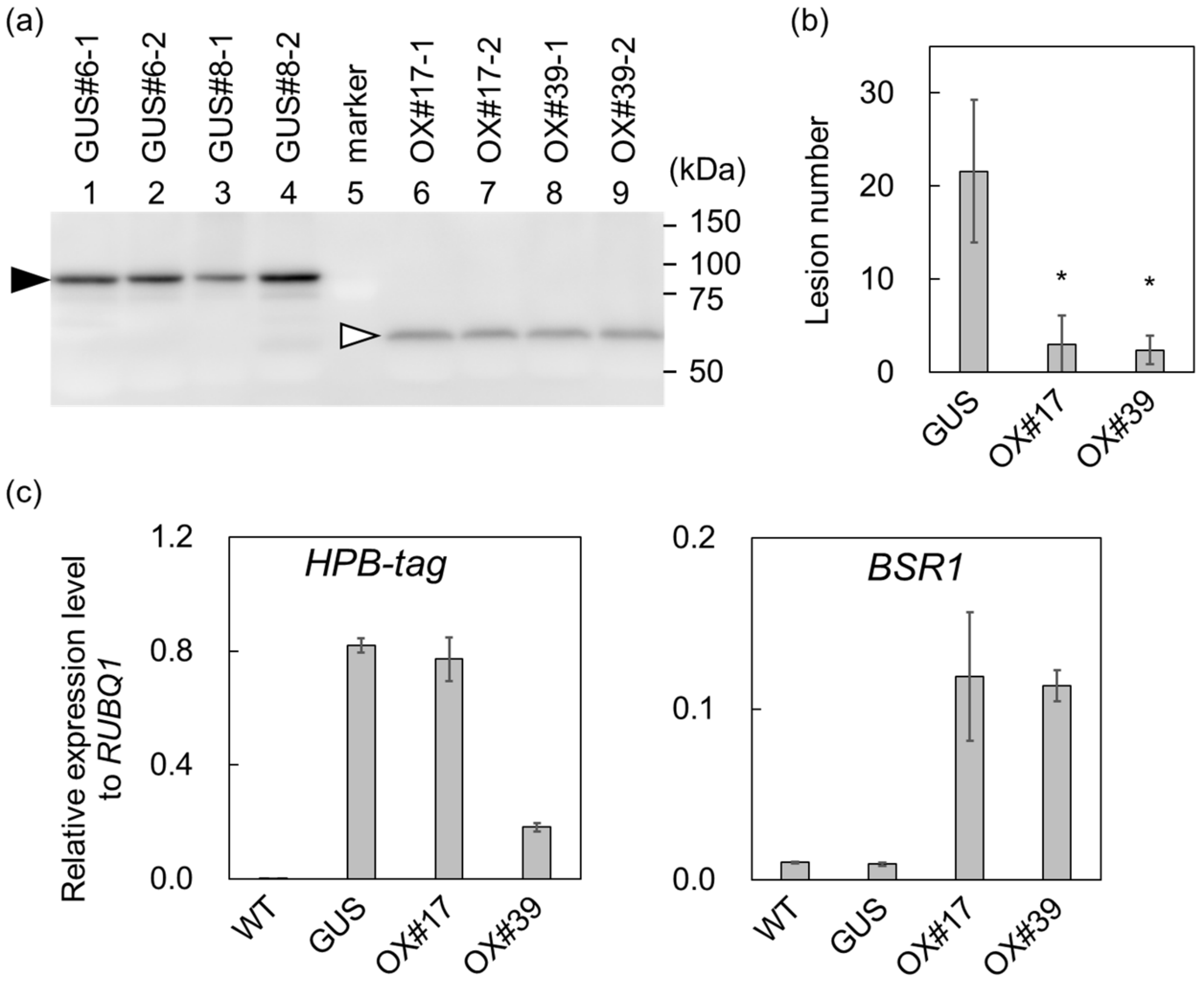

Figure S1. Preparation of rice lines overexpressing HPB-tagged proteins. (a) BSR1-HPB and GUS-HPB were detected at the predicted molecular sizes in western analyses of T1 plants. Black arrowhead, GUS-HPB (81.9 kDa); White arrowhead, BSR1-HPB (58.2 kDa). (b) The overexpression of BSR1-HPB conferred resistance to rice blast. A 5.8×10^5 ml^{-1} suspension of conidia was sprayed onto plants at the 5.5–6 leaf stage. The number of compatible lesions on the 6th leaf blades of each plant were calculated at 5 d after inoculation. Values are presented as the means ± standard deviations (n = 5). Asterisks indicate significant differences between the values of wild-type and other lines (Dunnett's test; $*p < 0.05$). (c) Transcript levels of HPB-tagged transgenes and BSR1 in suspension-cultured rice cells as assessed by qRT-PCR. Transcript levels were normalized against the *RUBQ1* internal control levels. Values are presented as the means ± standard deviations of three biological replicates. OX#17, BSR1-HPB:OX#17; OX#39, BSR1-HPB:OX#39; GUS, GUS-HPB:OX; WT, wild-type.

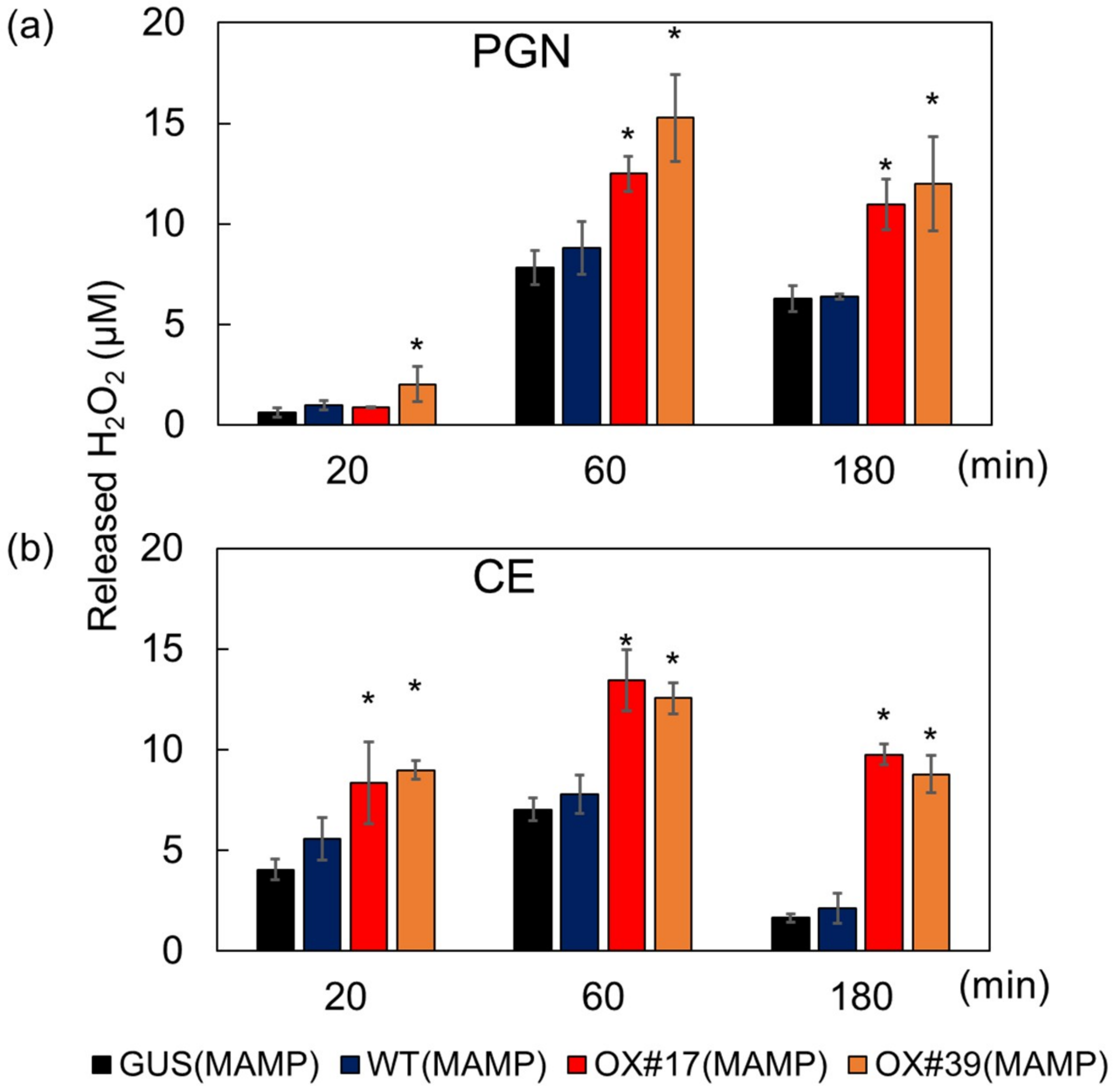

Figure S2. BSR1-HPB:OX cells produced greater amounts of H_2O_2 compared with GUS-HPB:OX cells in response to peptidoglycan (a) and chitin (b). The amount of H_2O_2 that was produced in a culturing tube during the experiment was calculated by subtracting the concentration at 0 min from that at the indicated times. Values are presented as the means ± standard deviations of three biological replicates. Asterisks indicate significant differences between the values of GUS-HPB:OX and those of other lines under the same conditions (Dunnett's test; $*p < 0.05$). PGN, peptidoglycan; CE, chitin elicitor; OX#17, BSR1-HPB:OX#17; OX#39, BSR1-HPB:OX#39; GUS, GUS-HPB:OX; WT, wild-type.

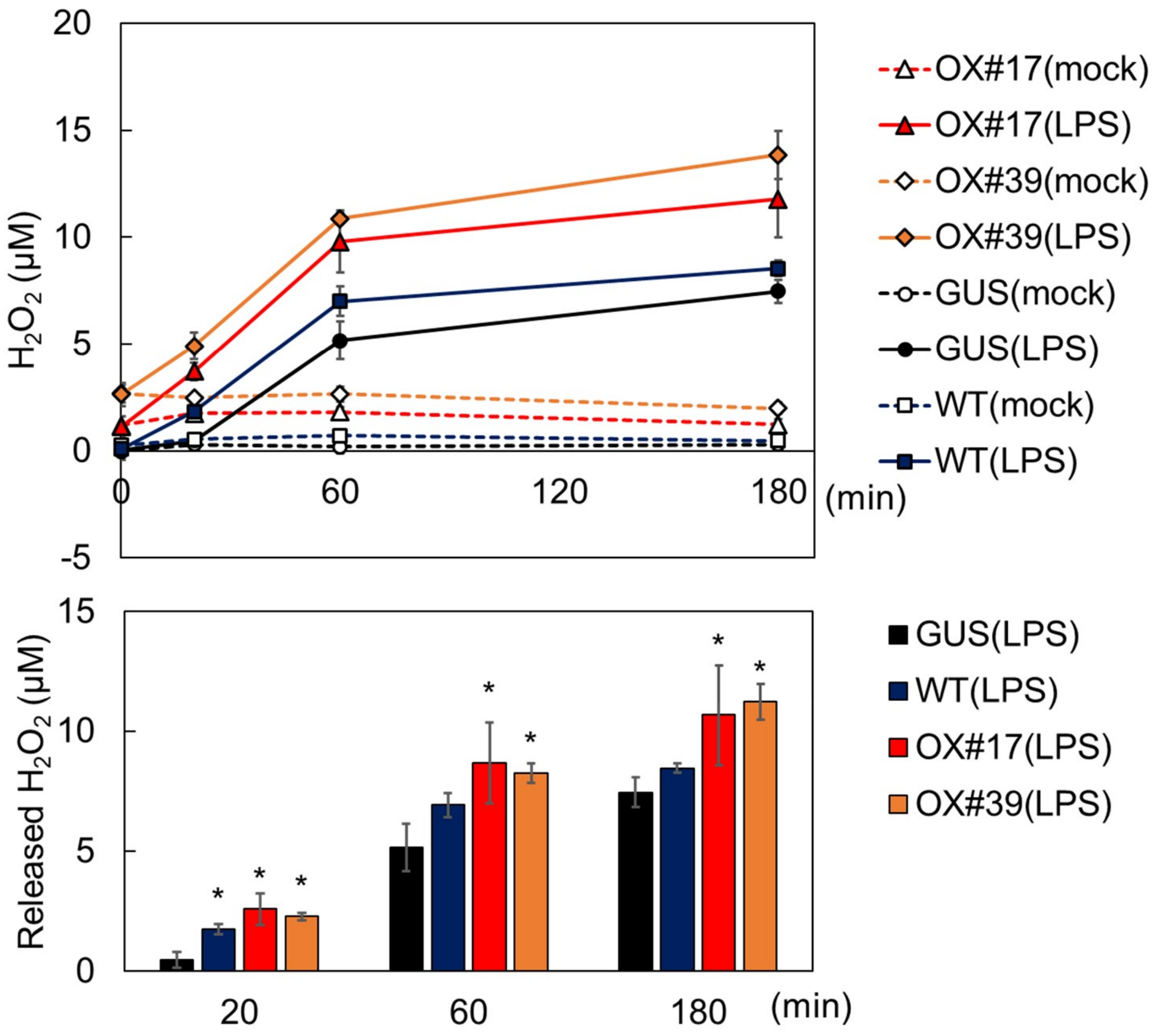

Figure S3. The overexpression of BSR1-HPB enhanced LPS-induced H_2O_2 production. H_2O_2 concentrations were measured before treatment and at 20, 60, and 180 min after treatment (a). The amount of H_2O_2 that was produced in a culturing tube during the experiment was calculated by subtracting the concentration at 0 min from that at the indicated times (b). Values are presented as the means ± standard deviations of three biological replicates. Asterisks indicate significant differences between the values at GUS-HPB:OX and those of other lines under the same conditions (Dunnett's test; $^*p < 0.05$). LPS, lipopolysaccharide; OX#17, BSR1-HPB:OX#17; OX#39, BSR1-HPB:OX#39; GUS, GUS-HPB:OX; WT, wild-type.

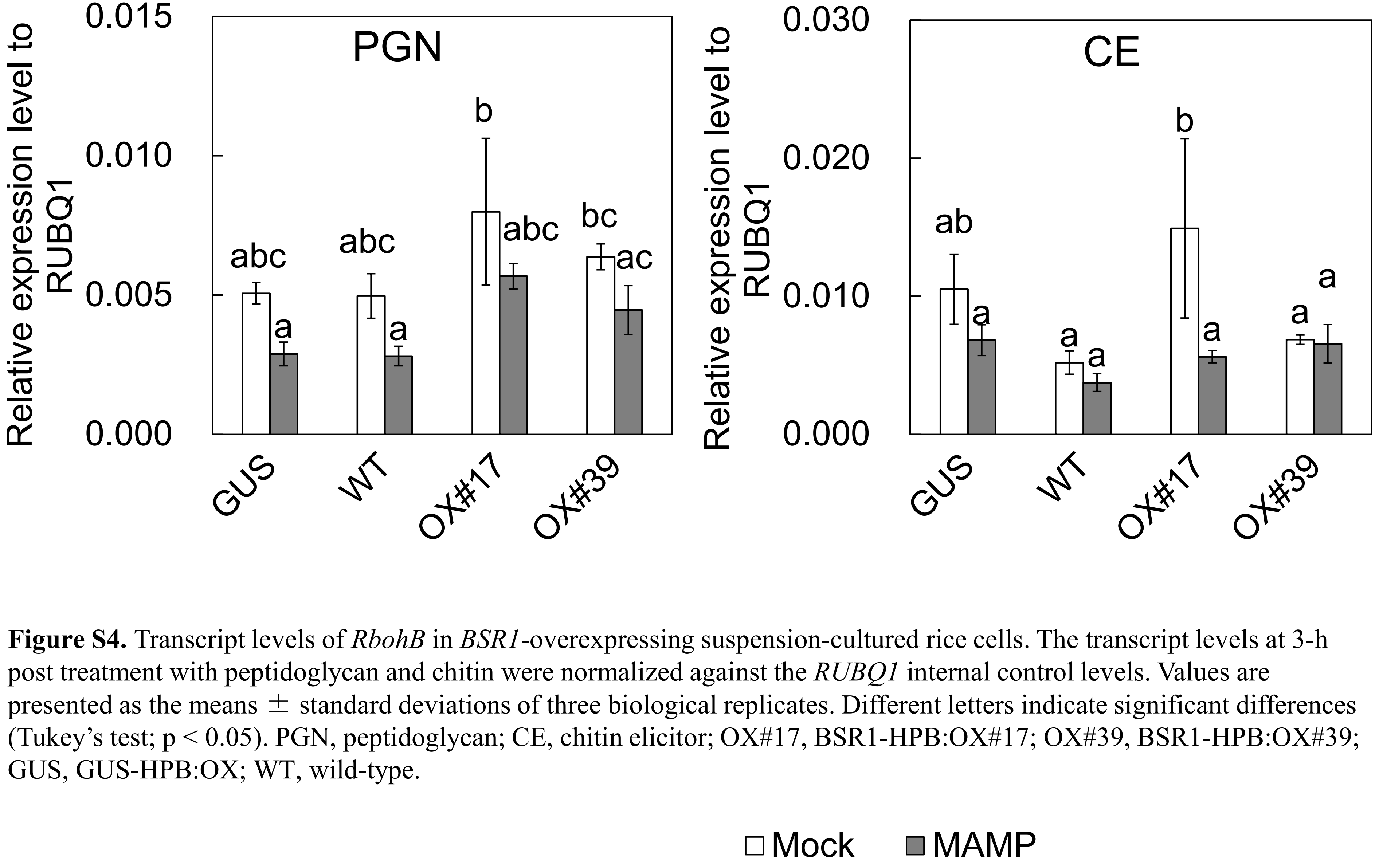

Figure S4. Transcript levels of *RbohB* in *BSR1*-overexpressing suspension-cultured rice cells. The transcript levels at 3-h post treatment with peptidoglycan and chitin were normalized against the *RUBQ1* internal control levels. Values are presented as the means ± standard deviations of three biological replicates. Different letters indicate significant differences (Tukey's test; $p < 0.05$). PGN, peptidoglycan; CE, chitin elicitor; OX#17, BSR1-HPB:OX#17; OX#39, BSR1-HPB:OX#39; GUS, GUS-HPB:OX; WT, wild-type.

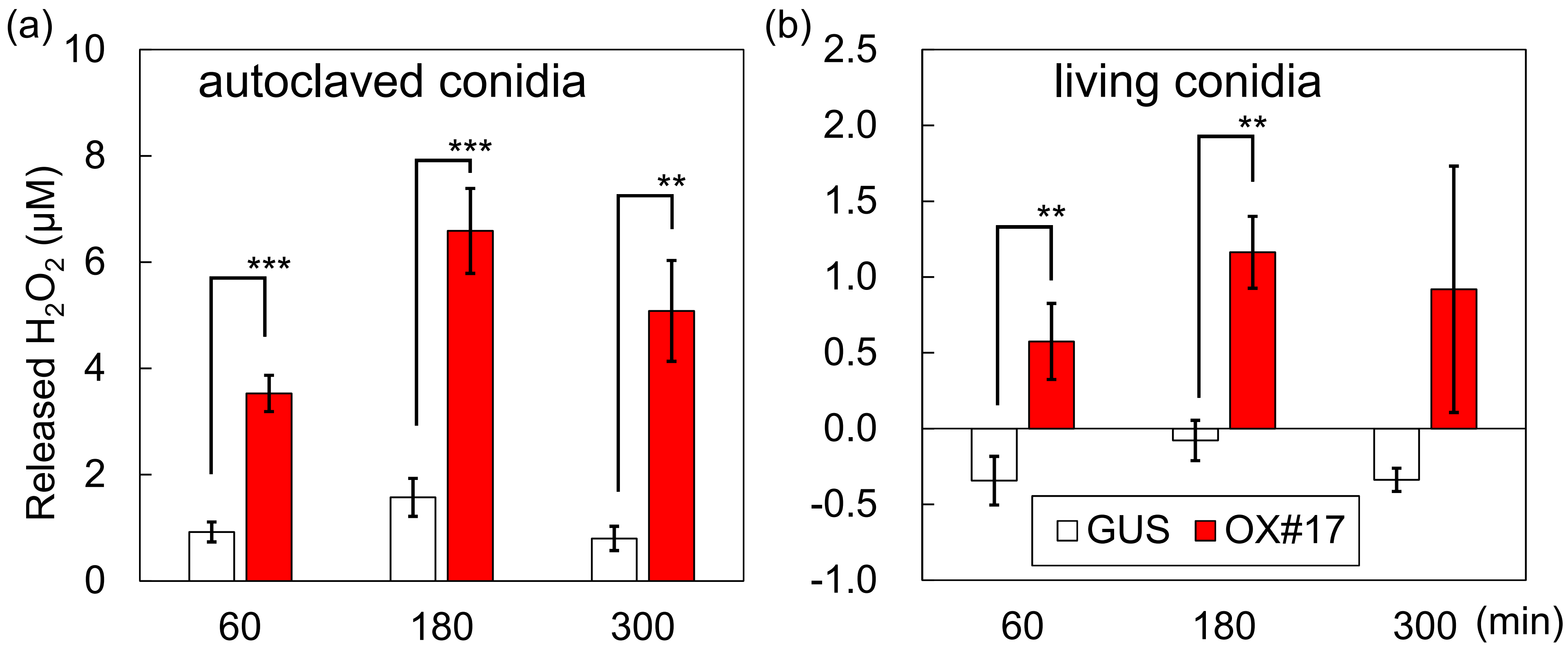

Figure S5. Changes in H_2O_2 concentrations between the untreated condition (0 min) and the indicated times. Leaf strips were cultivated with 8×10^4 ml^{-1} autoclaved conidia (a) or 8×10^3 ml^{-1} living conidia (b) in wells of a 12-well plate. The values were calculated by subtracting the concentration at 0 min from those at the indicated times and were presented as the means ± standard deviations of three biological replicates. Asterisks indicate significant differences between GUS-HPB:OX strips and BSR1-HPB:OX#17 strips (Student's t-test; $**p < 0.01$ and $***p < 0.001$). OX#17, BSR1-HPB:OX#17; GUS, GUS-HPB:OX.

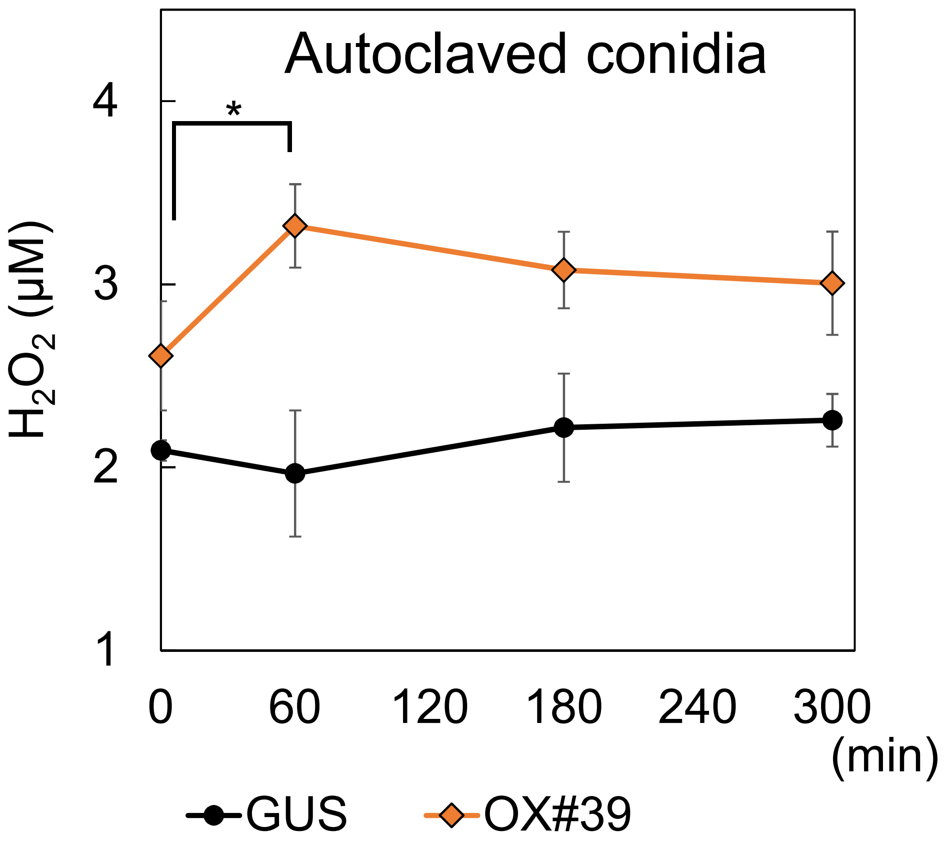

Figure S6. Rice leaf strips derived from BSR1-HPB:OX#39 plants caused an enhanced burst of H_2O_2 when exposed to autoclaved conidia of the blast fungus. Leaf strips were cultivated with 8×10^4 ml^{-1} autoclaved conidia in wells of a 12-well plate. H_2O_2 concentrations in wells were measured before treatment and at 60, 180, and 300 min after treatment. Values are presented as the means ± standard deviations of three biological replicates. Asterisks indicate significant differences between the untreated condition (0 min) values and the values at the indicated times in the same line (Student's *t*-test; $*p < 0.05$). Experiments were conducted two times with similar results. OX#39, BSR1-HPB:OX#39; GUS, GUS-HPB:OX.

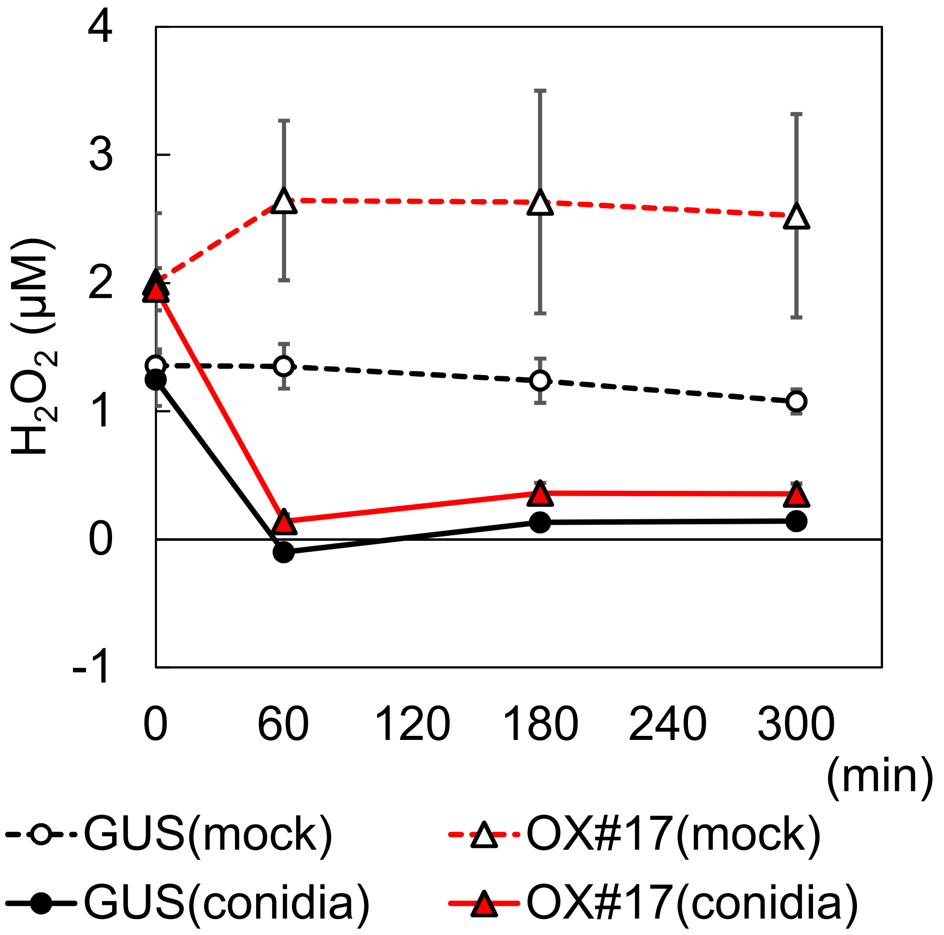

Figure S7. Blast fungus conidial suspensions showed ROS-degrading activities. Leaf strips were treated with 8×10^4 ml^{-1} living conidia and sterile water in wells of a 12-well plate. H_2O_2 concentrations in wells were measured before treatment and at 60, 180, and 300 min after treatment. Values are presented as the means ± standard deviations of three biological replicates. Experiments were conducted two times with similar results. OX#17, BSR1-HPB:OX#17; GUS, GUS-HPB:OX.

Table S1. List of primers used for qRT-PCR.

gene	primer 1	primer 2
PBZ1	CCGGCTTGGTCGACGACATT	CCGACTTTAGGACATGACTT
PAL1	GCTATCAACGAAGGCAAGCAC	GCCTCCACACTCCACTGTTATTC
KSL4	GTATTTCATGGGACAAAATCTCTGG	CCATCCTTGCATTCCCTCTC
DPF	CGTGCAAACCTAACATTACA	GGCACCTCCCTTTTTCTTCTT
RbohB	TCGGTGTGTTCTACTGTGGTGAG	CTTGTGTTTGTCTTGTGGGTGAA
HPB-tag	GCTCCGAAACATCATCACACC	TGCTCCATCTTCATTGCCTCT
BSR1	CCGGGACTTCAAAGCATCTAAC	TGTTGGTCCCTCCCTTGCT
RUBQ1	GGAGCTGCTGCTGTTCTAGG	TTCAGACACCATCAAACCAGA

Table S2. Statistical analysis of differences in H_2O_2 production among *BSR1*-knockout cells and wild-type cells after MAMP treatments.

(a) PGN

	20	60	180 (min)
KO#1	0.49 ± 0.12	1.05 ± 0.01 *	2.44 ± 0.15 ***
KO#2	0.28 ± 0.06 **	0.32 ± 0.08 ***	0.98 ± 0.22 ***
KO#8	0.34 ± 0.03 ***	1.20 ± 0.17 **	2.29 ± 0.21 ***
WT	0.63 ± 0.04	2.19 ± 0.22	4.70 ± 0.41

(b) LPS

	20	60	180 (min)
KO#1	0.26 ± 0.01	0.99 ± 0.04 ***	1.67 ± 0.24 *
KO#2	0.43 ± 0.07	1.11 ± 0.14 **	1.34 ± 0.16 **
KO#8	0.38 ± 0.07	1.10 ± 0.05 **	1.14 ± 0.15 **
WT	0.49 ± 0.11	1.79 ± 0.13	2.33 ± 0.26

(c) *Xoo*

	20	60	180 (min)
KO#1	2.78 ± 0.34 **	3.63 ± 0.07 *	4.19 ± 0.11 *
KO#2	2.03 ± 0.12 ***	2.50 ± 0.62 **	2.81 ± 0.34 *
KO#8	2.24 ± 0.26 ***	2.88 ± 0.10 *	2.54 ± 0.27 ***
WT	5.00 ± 0.45	6.04 ± 0.75	4.40 ± 0.06

Values are presented as the means ± standard deviations of three biological replicates, which are the same as the values for peptidoglycan (a), LPS (b), and autoclaved *X. oryzae* pv. *oryzae* (*Xoo*; c) treatments in Fig. 1. Asterisks indicate significant differences between the values at wild-type and those of other lines (Student's *t*-test; $*p < 0.05$, $**p < 0.01$, and $***p < 0.001$). PGN, peptidoglycan; LPS, lipopolysaccharide; KO#1, *bsr1-1*#13-1; KO#2, *bsr1-2*#16-2; KO#8, *bsr1-8*#5-1; WT, wild-type.
